# Biocontrol Efficacy of Microbial Antagonists Against *Pectobacterium carotovorum*-Induced Soft Rot in Fruits and Vegetables in East Asia: A Systematic Review and Meta-Analysis

**DOI:** 10.3390/foods15091580

**Published:** 2026-05-04

**Authors:** Habiba Lawal, Mohammed Sani Gaddafi, Esa Abiso Godana, Aasia Muhammed Jamiu, Abdulgaffar Usman El-Yakub, Gerefa Sefu Edo, Opoku Genevieve Fremah, Hongyin Zhang, Qiya Yang

**Affiliations:** 1 School of Food and Biological Engineering, Jiangsu University, Zhenjiang 212013, China; habeebalawal1@gmail.com (H.L.); esaabiso@gmail.com (E.A.G.); hasiatemjay@gmail.com (A.M.J.); sefgare@gmail.com (G.S.E.); gopoku@htu.edu.gh (O.G.F.); 2 Ministry of Animal Health, Husbandry, and Fisheries, Kebbi State, Birnin-Kebbi 860001, Nigeria; gaddafimohammedsani@yahoo.com; 3 Medical Biochemistry Department, Faculty of Basic Medical Sciences, Kaduna State University, Kaduna 800244, Nigeria; abdulgaffarusmane@gmail.com

**Keywords:** biocontrol, *Pectobacterium carotovorum*, soft rot, fruits and vegetables, East Asia, meta-analysis, disease incidence reduction, postharvest diseases

## Abstract

Soft rot disease caused by *Pectobacterium carotovorum* is a major postharvest problem in fruits and vegetables, particularly in East Asia. This systematic review and meta-analysis aimed to collate and critically evaluate studies on the biocontrol efficacy of microbial antagonists against *P. carotovorum* in East Asia, quantitatively estimate their effectiveness, and identify research gaps. A systematic search of PubMed, Web of Science, and Scopus databases was conducted following PRISMA guidelines and yielded 14 eligible studies (21 datasets) from East Asia. The overall pooled estimate of the relative reduction in disease incidence by microbial antagonists was 82% (95% CI: 74–88%; I^2^ = 88.2%), indicating high but highly variable biocontrol efficacy across studies. Subgroup analyses revealed varying efficacy among different antagonists, with Chitosan-enhanced *Lactobacillus pentosus* and *Leuconostoc fallax* showing the highest potential (100% reduction), followed by *Pseudomonas aeruginosa* (90%), *Bacillus velezensis* (85.7%), and *Lactobacillus paracasei* WX322 (82.2%). Meta-regression identified BCA cell concentration, sample size, storage days, and storage temperature as significant sources of heterogeneity. Most studies were conducted in China, highlighting the need for more research in other East Asian countries. Microbial antagonists demonstrate substantial potential for reducing soft rot incidence, yet the high heterogeneity across studies warrants cautious interpretation of the pooled effect. While these findings are promising, further field validation and expanded geographical representation are needed.

## 1. Introduction

Soft rot disease caused by *Pectobacterium carotovorum*, a Gram-negative, facultatively anaerobic bacterium belonging to the family *Pectobacteriaceae*, remains one of the most devastating postharvest bacterial infections in fruits and vegetables globally [1,2]. Characterized by tissue maceration, foul odor, and rapid spoilage, *P. carotovorum*-induced soft rot significantly undermines the quality, marketability, and shelf-life of fresh produce such as eggplants, tomatoes, cucumbers, carrots, and potatoes [3,4]. This pathogen thrives under high humidity and temperature conditions typical of many storage and distribution environments, accelerating postharvest decay and posing serious challenges to food security and the agricultural economy, particularly in developing and emerging economies [5,6,7].

Globally, postharvest losses in fruits and vegetables are estimated to range between 30 and 50% [8,9], with bacterial soft rot being a major contributor [10,11,12]. In East Asia, a region characterized by dense agricultural activity, extensive fruit and vegetable consumption, and warm climatic conditions, the impact of *P. carotovorum* is notably severe [13,14]. Countries such as China, Japan, South Korea, and Vietnam consistently report substantial economic losses from soft rot infections during storage, transportation, and marketing [15,16]. The pathogen’s aggressive lytic enzymes, including pectate lyases and cellulases, degrade plant cell walls, causing liquefaction and rot, often rendering large quantities of produce unfit for sale or consumption [17,18]. The situation is exacerbated by limited cold-chain infrastructure in parts of East Asia and the rising resistance to conventional chemical bactericides [19,20,21].

Conventional management strategies for soft rot have relied heavily on synthetic bactericides and postharvest handling interventions [22,23]. However, growing concerns about chemical residues, environmental safety, and the emergence of antimicrobial resistance necessitate the exploration of safer, sustainable, and eco-friendly alternatives [24]. In this context, microbial antagonists, such as species of *Bacillus*, *Pseudomonas*, *Trichoderma*, and *Aureobasidium*, have emerged as promising biocontrol agents for the management of postharvest soft rot [25,26]. These antagonists exhibit diverse modes of action, including competitive exclusion, antibiosis, induction of host resistance, and nutrient sequestration, making them attractive candidates for integrated postharvest disease management [27,28].

Microbial antagonists employ diverse and often complementary mechanisms to suppress postharvest pathogens [29]. These include antibiosis (production of antimicrobial metabolites such as lipopeptides, bacteriocins, and volatile organic compounds), competitive exclusion (rapid colonization of wound sites and nutrient depletion), induced systemic resistance (activation of plant defense pathways, including reactive oxygen species and pathogenesis-related proteins), and quorum quenching (disruption of pathogen cell-to-cell communication). This review encompasses both bacterial and fungal microbial antagonists, including *Trichoderma* spp., as biocontrol agents against *P. carotovorum*. While *Trichoderma* is conventionally used against fungal pathogens, recent evidence demonstrates its efficacy against bacterial soft rot via antibiosis, competition, and host defense induction [29,30].

Despite a growing body of experimental evidence supporting the efficacy of microbial antagonists against *P. carotovorum*, findings remain fragmented and context-specific. There is a lack of consolidated data that could inform regional policy, guide research investment, and support the practical adoption of microbial biocontrol strategies in East Asia. To date, no prior systematic review or meta-analysis has quantitatively synthesized the efficacy of microbial antagonists against *P. carotovorum*-induced soft rot specifically in East Asia. Therefore, a systematic review and meta-analysis are warranted to synthesize the available evidence, quantify the effectiveness of microbial antagonists across different fruit and vegetable hosts, and identify critical gaps in research and application. This work seeks to provide the first region-specific, robust, evidence-based quantitative assessment of the biocontrol potential of microbial antagonists against *P. carotovorum*-induced soft rot, with a regional focus on East Asia.

The objectives of this systematic review and meta-analysis are threefold: (1) to collate and critically evaluate existing studies on the biocontrol efficacy of microbial antagonists against *P. carotovorum* in fruits and vegetables within East Asia; (2) to quantitatively estimate the effectiveness of these antagonists through meta-analysis; and (3) to identify methodological limitations, and future directions for improving postharvest disease management in the region. By synthesizing this data, this study aims to provide actionable insights for scientists, policymakers, and agricultural practitioners seeking sustainable solutions to postharvest losses, thereby enhancing food security, farmer income, and environmental safety in East Asia.

## 2. Methods

### 2.1. Study Design and Search Strategies

This systematic review was conducted and reported in accordance with the Preferred Reporting Items for Systematic Reviews and Meta-Analyses (PRISMA, 2020) guidelines (Supplementary S1) [31]. However, a search protocol was designed and used for this work. To identify relevant papers, the PubMed, Web of Science, and Scopus databases were systematically searched in accordance with PRISMA recommendations. The keywords were applied using Boolean operators (AND, OR); for example, the Web of Science search used: TS = (“*P. carotovorum*” OR “*Erwinia carotovora*” OR “soft rot” OR “postharvest soft rot”) AND (“biocontrol” OR “biological control” OR “antagonistic bacteria” OR “bacterial antagonist” OR “biological antagonism”) AND (“fruit” OR “vegetable” OR “postharvest disease” OR “crop disease control”) AND (“East Asia” OR “China” OR “Japan” OR “South Korea” OR “North Korea” OR “Mongolia” OR “Taiwan”) SCOPUS = TITLE-ABS-KEY = (“*P. carotovorum*” OR “*E. carotovora*” OR “soft rot” OR “postharvest soft rot”) AND (“biocontrol” OR “biological control” OR “antagonistic bacteria” OR “bacterial antagonist” OR “biological antagonism”) AND (“fruit” OR “vegetable” OR “postharvest disease” OR “crop disease control”) AND (“East Asia” OR “China” OR “Japan” OR “South Korea” OR “North Korea” OR “Mongolia” OR “Taiwan”) PUBMED = ((“*P. carotovorum*” OR “*E. carotovora*” OR “soft rot” OR “postharvest soft rot”) AND (“biocontrol” OR “biological control” OR “antagonistic bacteria” OR “bacterial antagonist” OR “biological antagonism”) AND (“fruit” OR “vegetable” OR “postharvest disease” OR “crop disease control”)) The search was limited to titles and abstracts. Filters restricted results to documents in English and the publication type “Original article.” We deliberately excluded abstracts, preprints, and theses to maintain rigorous methodological quality, guarantee the availability of complete study details, and ensure the reliability of reporting. This choice, while it may restrict the inclusion of unpublished data, effectively mitigates the risk of incorporating studies that have not undergone formal peer review. The search was executed between February 10 and 13, 2025, yielding 191 articles. The reference lists of these identified papers were also screened to locate additional studies eligible for inclusion. Relative disease incidence reduction (RDIR) was defined as the proportion of fruits/vegetables with increased shelf-life when treated with microbial antagonist/biocontrol agents (BCA) either singly or in combination with enhancers.

$$RDIR=\frac{Disease\;incidence\;in\;pathogen-BCA-treated\;disease\;incidence}{Number\;of\;total\;fruits/vegetables\;exposed\;to\;the\;isolate}$$

### 2.2. Study Eligibility Criteria and Data Extraction

All identified references were exported to EndNote X (version 20.6, Clarivate Analytics) to facilitate efficient reference management. The “Find Duplicate” function was utilized to automatically identify and remove duplicate records, followed by a thorough manual review to ensure completeness. Subsequently, the remaining references were transferred to Microsoft Excel, where two independent reviewers (GMS and HL) evaluated and screened the titles and abstracts of the studies using a standardized eligibility form for data extraction. The same reviewers independently performed both full-text assessment and data extraction, and any discrepancies were resolved by discussion or consulting a third reviewer (EAG). Studies with missing critical variables (e.g., disease incidence, sample size) were excluded. The extracted data included the authors’ names, publication year, study country, sample size, pathogen strain, crop type, antagonistic bacteria used, study design, storage temperature, storage duration, pathogen concentration (CFU/mL), concentration of the antagonistic bacteria, and disease incidence.

### 2.3. Inclusion and Exclusion Criteria

#### 2.3.1. Study Inclusion Criteria

Studies were eligible and included in this study if the:

- Studies were conducted in East Asian countries.
- Studies specifically investigating *P. carotovorum*.
- Studies evaluate the efficacy of bacterial antagonists as a biocontrol agent.
- Studies involved fruits/vegetables affected by *P. carotovorum*-induced soft rot.
- Studies used either in vitro, in vivo or under field/postharvest conditions as study design.
- Study is original research article presenting data with measurable biocontrol efficacy outcomes.
- Studies are published in English language and accessible in full-text, regardless of the year of publication.

#### 2.3.2. Study Exclusion Criteria

Studies were not eligible and excluded if:

- Conducted outside East Asia or with unclear study location not attributed to any East Asian country.
- Studies that focus on other microbial antagonists other than bacteria for soft rot control.
- Studies targeting other soft rot pathogens other than *P. carotovorum*.
- Literature reviews, meta-analyses, opinion pieces, conference abstracts, or studies that do not report measurable efficacy bio-control outcomes.
- Studies are not published in the English Language and the full text is not accessible.
- Studies involved fruits/vegetables but are not affected by *P. carotovorum*-induced soft rot.

### 2.4. Study Quality Assessment

To assess the methodological quality of the included studies, the review employed the Joanna Briggs Institute (JBI) critical appraisal checklists tool, designed specifically to fit each study’s design (Supplementary S2). To facilitate interpretation and comparison across studies, quality scores were assigned based on JBI standard scoring categorization. Studies that met at least 7–9 of the applicable criteria were classified as high quality, while those scoring between 5 and 6 were rated as moderate quality studies. Studies with scores below 5 were categorized as low quality. These cutoff points were established to differentiate studies with minimal risk of bias from those with significant methodological issues, while also ensuring that overly strict exclusion criteria did not prevent the inclusion of relevant research in a field with limited available evidence.

### 2.5. Geographical Distribution

The geographical spread of the studies was represented through descriptive mapping methods. Information at the country level was gathered from included studies and mapped based on study locations using QGIS version 3.44, utilizing publicly available country shapefiles boundaries obtained from Natural Earth. The resulting maps illustrate the distribution of study sites and the reported BCA outcomes across East Asia. This map serves solely for visualization and descriptive purposes. It is important to note that no analysis was conducted on the map.

### 2.6. Data Analysis

All statistical analyses were conducted using OpenMeta [Analyst] and Comprehensive Meta-Analysis (CMA) version 3.0 [32]. Given the substantial between-study heterogeneity anticipated in this field, a random-effects modelling framework was adopted throughout. Although the DerSimonian–Laird (DL) estimator was initially explored for comparison, it was not retained for reporting due to its known limitations in handling high heterogeneity. Therefore, all primary, subgroup, and Meta-regression analyses presented in this study were performed using the Restricted Maximum Likelihood (REML) method, which provides more robust and less biased estimates of between-study variance under such conditions. The effect size was defined as the relative disease incidence reduction (RDIR), with proportions logit-transformed prior to analysis and back-transformed for interpretation. Statistical heterogeneity was assessed using the I^2^ statistic, with values above 75% considered indicative of substantial heterogeneity.

Subgroup analyses were conducted using the same REML framework to ensure methodological consistency and were stratified based on biologically and methodologically relevant variables, including type of biocontrol agent, crop type, and country of study. Meta-regression analyses were also performed using REML to evaluate the influence of continuous moderators such as sample size, storage duration, storage temperature, and microbial concentration.

Publication bias was assessed through visual inspection of funnel plots and formally tested using Egger’s regression test [33], with *p* < 0.05 considered indicative of potential small-study effects.

#### Meta-Analysis and Meta-Regression

The primary outcome measure was the relative disease incidence reduction (RDIR), expressed as a proportion. Subgroup analyses were conducted within the REML framework to explore potential sources of heterogeneity across categorical variables, including type of biocontrol agent, crop type, country, and study design. These subgroup variables were selected based on biological relevance and methodological differences that could influence treatment efficacy. Meta-regression analyses were performed using REML to evaluate the influence of continuous moderators, including microbial antagonist concentration, pathogen concentration, storage duration, storage temperature, and sample size. All statistical tests were two-sided, and statistical significance was set at *p* < 0.05.

## 3. Results

### 3.1. Systematic Literature Review Process and Study Characteristics

**Figure 1:**
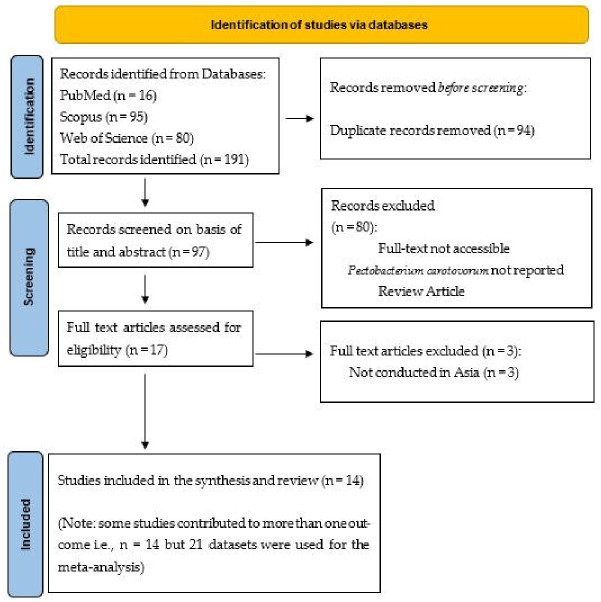
PRISMA flow diagram of the study selection process for inclusion in the review.

A total of 191 publications were retrieved from different databases. Duplicates were detected and removed in EndNote X (Version 20.6), leaving 97 publications for title and abstract screening. A further 80 records were excluded during title and abstract screening based on exclusion criteria. Fourteen eligible studies (21 datasets) were assessed for full-text screening for inclusion into the study (Figure 1).

**Figure 2:**
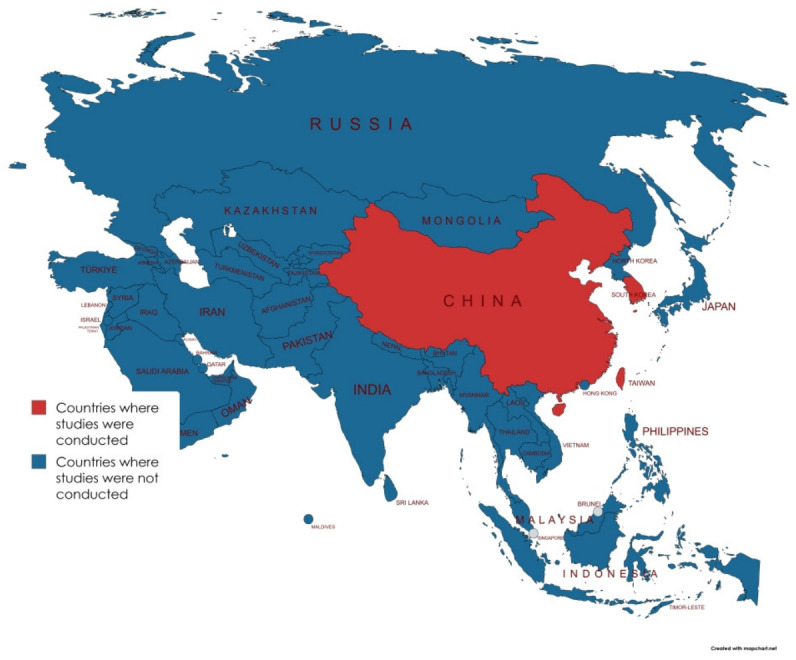
Geographical distribution of studies conducted in East Asia showing the countries where studies were conducted.

Among the 14 included studies, 10 employed both in vitro and in vivo experimental study designs, one study utilized in vitro, in vivo, and field experiments, and two studies used in vitro and greenhouse experimental designs. In contrast, only one study employed a combination of in vitro and pot experiments as its study design (Table 1). All the reviewed studies were conducted in three East-Asian countries. Most of the studies (11) were conducted in China, two of the reviewed studies were reported from South Korea, and only one study was reported from Taiwan (Figure 2). A large number of publications were observed between 2018 and 2025. Four of the reviewed studies reported the incidence of soft rot caused by *P. carotovorum* on green pepper, three studies each reported on Chinese cabbage and tomatoes, two studies were conducted on cucumber, while only one study each was conducted on eggplant, radish, pinellia ternata, kimchi cabbage, and lettuce. Most of the reviewed studies used different antagonistic bacteria to demonstrate their potential role as biocontrol agents to reduce the effect of *P. carotovorum* on various crops in East Asia. *B. velezensis* was used on eggplant and Chinese cabbage to reduce the incidence of soft rot caused by *P. carotovorum, P. aeruginosa* was used on pinellia ternate plant to reduce the incidence of soft rot, *B. subtilis* were used on green pepper, chinese cabbage, and lettuce plant to reduce the incidence of soft rot, bacteriophage was used on kimchi cabbage to reduce the soft rot incidence, *Myxococcus xanthus* was used on calla lily to reduce soft rot incidence, *L. paracasei* was used on green pepper, Chinese cabbage, cucumber, tomato and green bean to reduce the incidence of soft rot. *Trichoderma asperellum* was used on Chinese cabbage, cherry tomato, and carrot to reduce the incidence of soft rot, *Bacillus amyloliquefaciens* was used on tomato to reduce soft rot incidence (Table 1).

The systematic review showed chitosan enhanced *L. pentosus* and *L. fallax* (100%) has the most potential as a biocontrol for reduction in postharvest soft rot disease incidence caused by *P. carotovorum,* followed by *P. aeruginosa* (90%), *B. velezensis* (85.7%), *L. paracasei* WX322 (82.2%) while *B. subtilis* (55%) showed lower potential to reduce postharvest soft rot disease caused by *P. carotovorum* (Table 1).

### 3.2. Risk of Bias and Assessment of Study Quality

The methodological rigor and risk of bias of all 14 included studies was evaluated through the application of the Joanna Briggs Institute (JBI) critical appraisal checklist tool for estimating studies using nine criteria. The JBI tool consist of questions with “Yes”, “No”, “Unclear” or “Not applicable” response. Scores are allocated as 1 for “Yes” and 0 for “No”. Extracted studies were classified as high, medium or low quality if the summed score per study were 7–9, 5–6, and 0–4, respectively. In accordance with the predefined scoring criteria, all the studies fulfilled the threshold for high methodological quality/low risk of bias (scores 7–9). Studies attaining high-quality designations were characteristically distinguished by clearly delineated sampling frameworks, statistically adequate sample sizes, comprehensive methodological documentation, and the consistent application of internationally recognized laboratory protocols for the determination of relative disease incidence reduction efficacy of microbial antagonists on fruits and vegetables.

To evaluate the robustness and reliability of the pooled RDIR estimate, a sensitivity analysis was performed using the leave-one-out statistical meta-analysis method. The pooled RDIR estimate remained largely unchanged compared with the primary analysis, and substantial between-study heterogeneity persisted (I^2^ = 88.2%, *p* < 0.001). These findings substantiate that the overall pooled RDIR estimate was not disproportionately influenced by studies with an elevated risk of bias, thereby affirming the stability and methodological robustness of the meta-analytic results.

**Figure 3:**
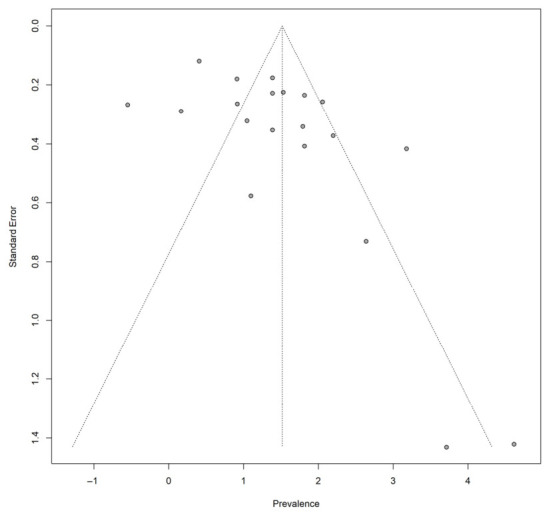
Funnel plot assessing publication bias for studies reporting Biocontrol Efficacy of Microbial Antagonists Against *Pectobacterium carotovorum*-Induced Soft Rot (Egger’s t = 2.41, df = 19, *p* = 0.0262) in East Asia [1,10,31,34,35,36,37,38,39,40,41,42,43,44].

A linear regression test of funnel plot asymmetry (Egger’s test) indicated statistically significant asymmetry (t = 2.41, df = 19, *p* = 0.0262), suggesting the presence of small-study effects. The estimated bias coefficient was 3.15 (SE = 1.31), indicating that smaller studies tended to report higher effect sizes. However, given the substantial between-study heterogeneity (I^2^ = 88.2%), the observed asymmetry may reflect both genuine variability in study outcomes and potential publication bias (Figure 3). Therefore, the results should be interpreted with caution.

### 3.3. Meta-Analysis and Meta-Regression

**Figure 4:**
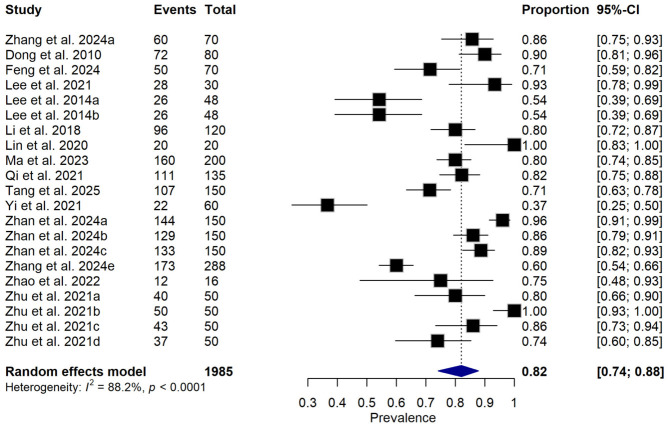
Forest plot showing subgroup analysis of relative disease incidence reduction rate (RDIR) of Microbial Antagonists Against *Pectobacterium carotovorum*-Induced Soft Rot in fruits and vegetables. The pooled estimate was derived using a random-effects meta-analysis model. Squares (black) represent individual study estimates, with sizes proportional to the inverse variance weighting, while horizontal lines indicate 95% confidence intervals (CI). The diamond (blue) represents the pooled of relative disease incidence reduction estimate. I^2^ indicates the degree of between-study heterogeneity. Total number of studies: 14; total sample size: 1985 [1,10,31,34,35,36,37,38,39,40,41,42,43,44].

The overall pooled estimate of relative disease incidence reduction (RDIR) was 82% (95% CI: 74–88%) (Figure 4), indicating a high level of disease suppression across the included studies. However, substantial heterogeneity was observed (I^2^ = 88.2%, *p* < 0.001), suggesting considerable variability in effect sizes across different experimental conditions. Given this high heterogeneity, the pooled estimate should be interpreted as a descriptive summary rather than a universally generalizable effect size.

**Figure 5:**
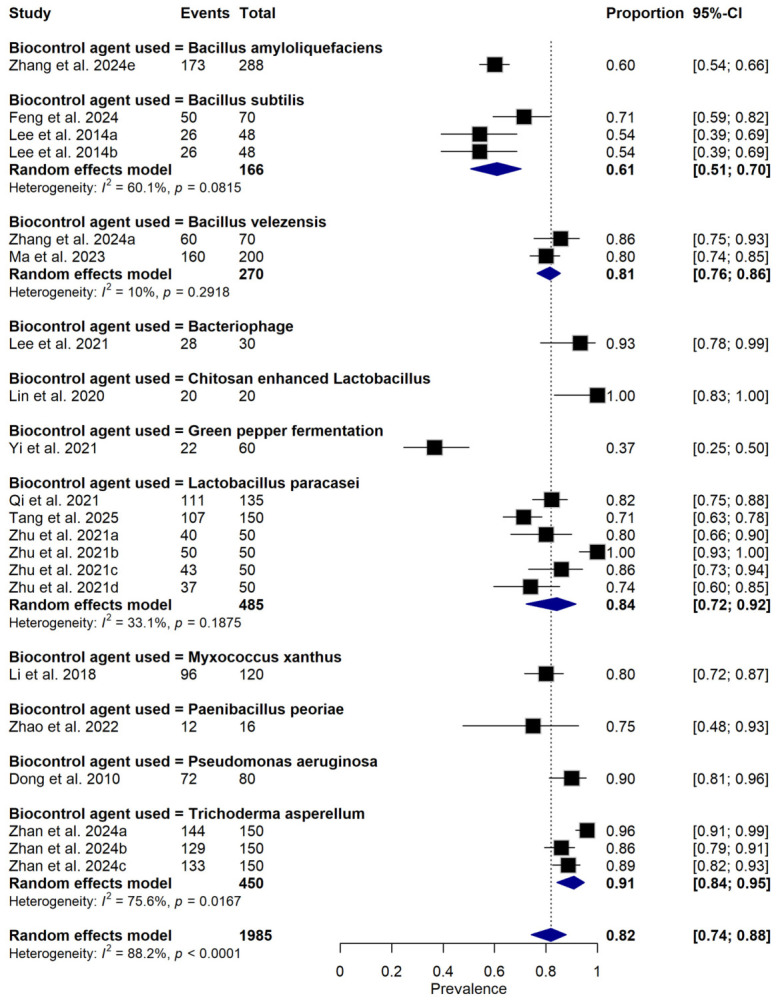
Forest plot showing subgroup analysis of relative disease incidence reduction rate (RDIR) of Microbial Antagonists Against *Pectobacterium carotovorum*-Induced Soft Rot in fruits and vegetables based on the type of biocontrol agent used. The pooled estimate was derived using a random-effects meta-analysis model. Squares (black) represent individual study estimates, with sizes proportional to the inverse variance weighting, while horizontal lines indicate 95% confidence intervals (CI). The diamond (blue) represents the pooled of relative disease incidence reduction estimate. I^2^ indicates the degree of between-study heterogeneity. Total number of studies: 14; total sample size: 1985 [1,10,31,34,35,36,37,38,39,40,41,42,43,44].

**Figure 6:**
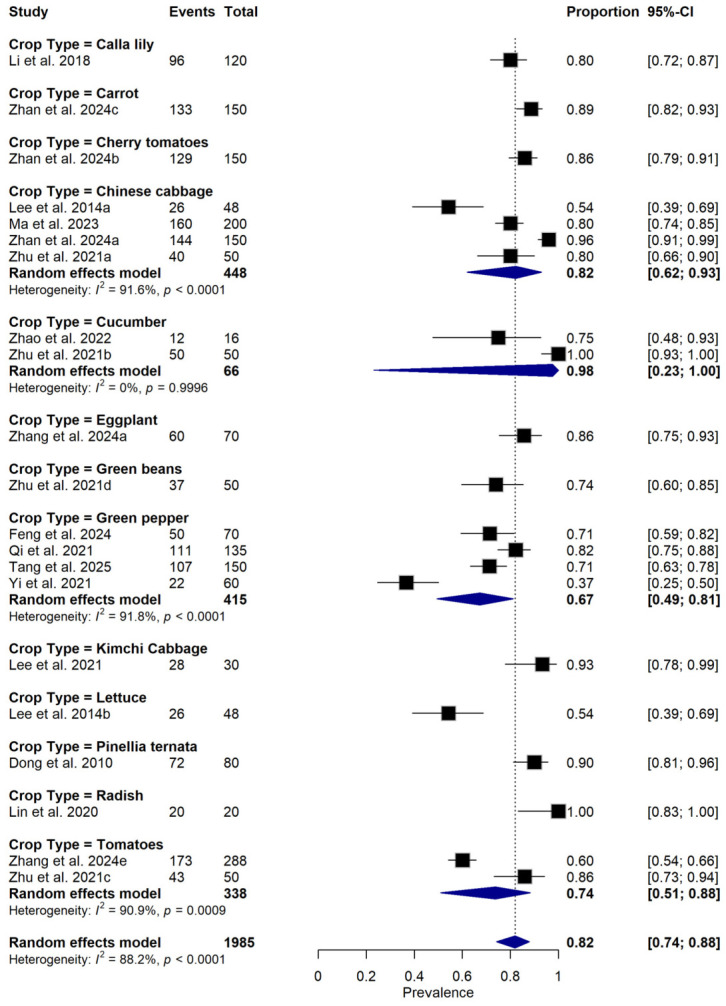
Forest plot illustrating subgroup analysis of relative disease incidence reduction rate (RDIR) of Microbial Antagonists Against *Pectobacterium carotovorum* stratified by crop type. The pooled estimate was derived using a random-effects meta-analysis model. Squares (black) represent individual study estimates, with sizes proportional to the inverse variance weighting, while horizontal lines indicate 95% confidence intervals (CI). The diamond (blue) represents the pooled prevalence estimate. I^2^ indicates the degree of between-study heterogeneity. Total number of studies: 14; total sample size: 1985 [1,10,31,34,35,36,37,38,39,40,41,42,43,44].

**Figure 7:**
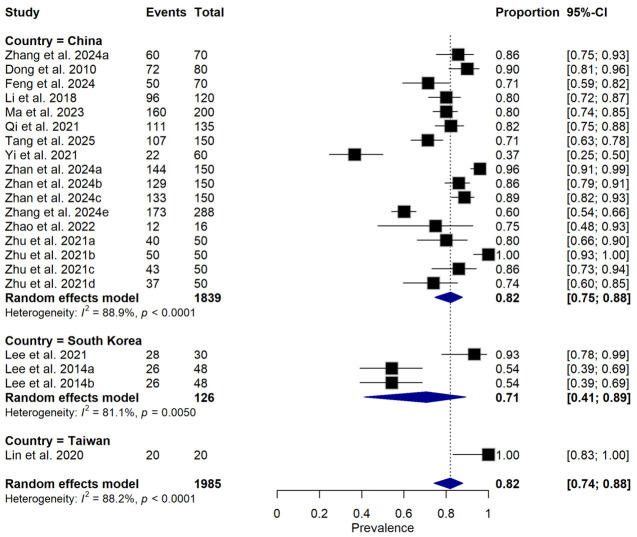
Forest plot illustrating subgroup analysis of relative disease incidence reduction rate (RDIR) of Microbial Antagonists Against *Pectobacterium carotovorum* stratified by country. The pooled estimate was derived using a random-effects meta-analysis model. Squares (black) represent individual study estimates, with sizes proportional to the inverse variance weighting, while horizontal lines indicate 95% confidence intervals (CI). The diamond (blue) represents the pooled prevalence estimate. I^2^ indicates the degree of between-study heterogeneity. Total number of studies: 14; total sample size: 1985 [1,10,31,34,35,36,37,38,39,40,41,42,43,44].

**Figure 8:**
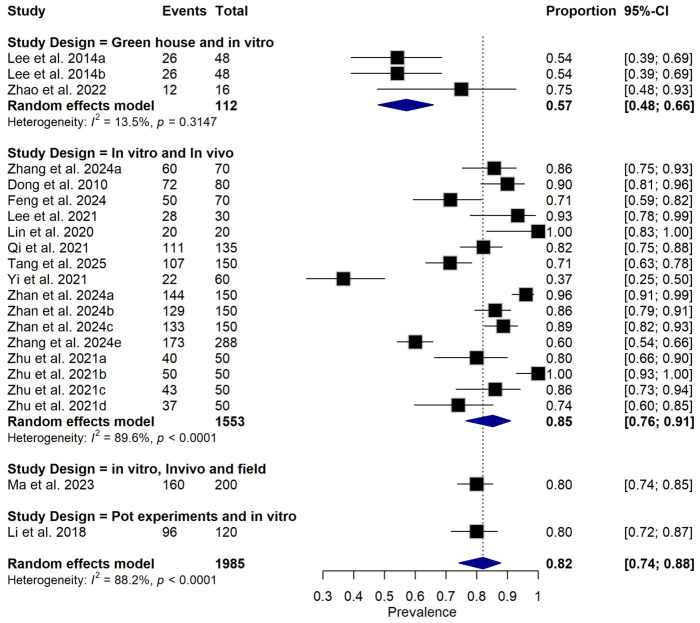
Forest plot illustrating subgroup analysis of relative disease incidence reduction rate (RDIR) of Microbial Antagonists Against *Pectobacterium carotovorum* stratified by study design. The pooled estimate was derived using a random-effects meta-analysis model. Squares (black) represent individual study estimates, with sizes proportional to the inverse variance weighting, while horizontal lines indicate 95% confidence intervals (CI). The diamond (blue) represents the pooled prevalence estimate. I^2^ indicates the degree of between-study heterogeneity. Total number of studies: 14; total sample size: 1985 [1,10,31,34,35,36,37,38,39,40,41,42,43,44].

**Figure 9:**
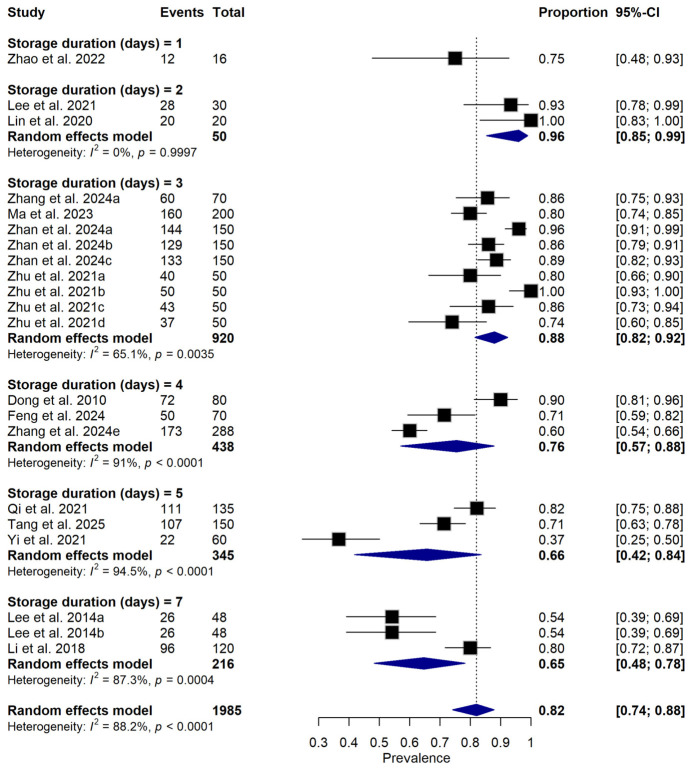
Forest plot illustrates subgroup analysis of relative disease incidence reduction rate (RDIR) of Microbial Antagonists Against *Pectobacterium carotovorum* stratified by storage duration. The pooled estimate was derived using a random-effects meta-analysis model. Squares (black) represent individual study estimates, with sizes proportional to the inverse variance weighting, while horizontal lines indicate 95% confidence intervals (CI). The diamond (blue) represents the pooled prevalence estimate. I^2^ indicates the degree of between-study heterogeneity. Total number of studies: 14; total sample size: 1985 [1,10,31,34,35,36,37,38,39,40,41,42,43,44].

**Figure 10:**
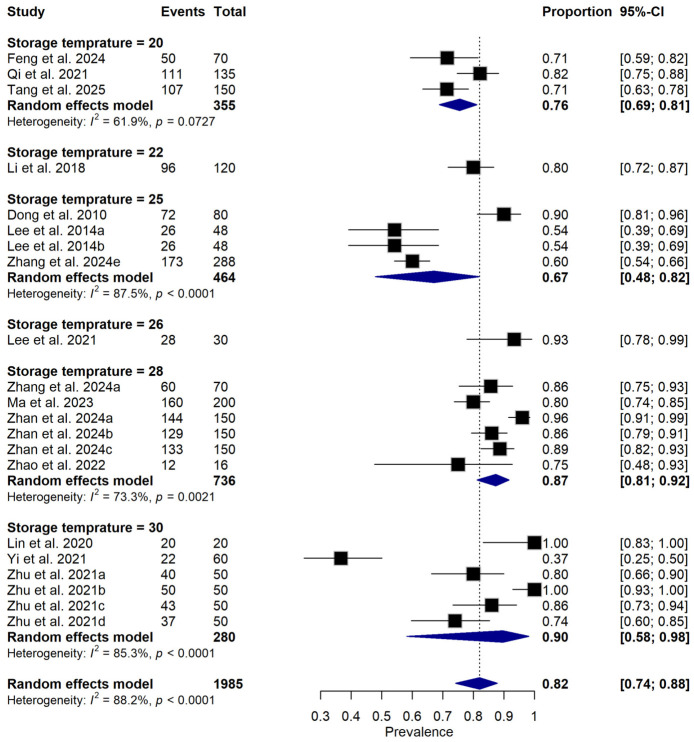
Forest plot illustrates subgroup analysis of relative disease incidence reduction rate (RDIR) of Microbial Antagonists Against *Pectobacterium carotovorum* stratified by storage temperature. The pooled estimate was derived using a random-effects meta-analysis model. Squares (black) represent individual study estimates, with sizes proportional to the inverse variance weighting, while horizontal lines indicate 95% confidence intervals (CI). The diamond (blue) represents the pooled prevalence estimate. I^2^ indicates the degree of between-study heterogeneity. Total number of studies: 14; total sample size: 1985 [1,10,31,34,35,36,37,38,39,40,41,42,43,44].

**Figure 11:**
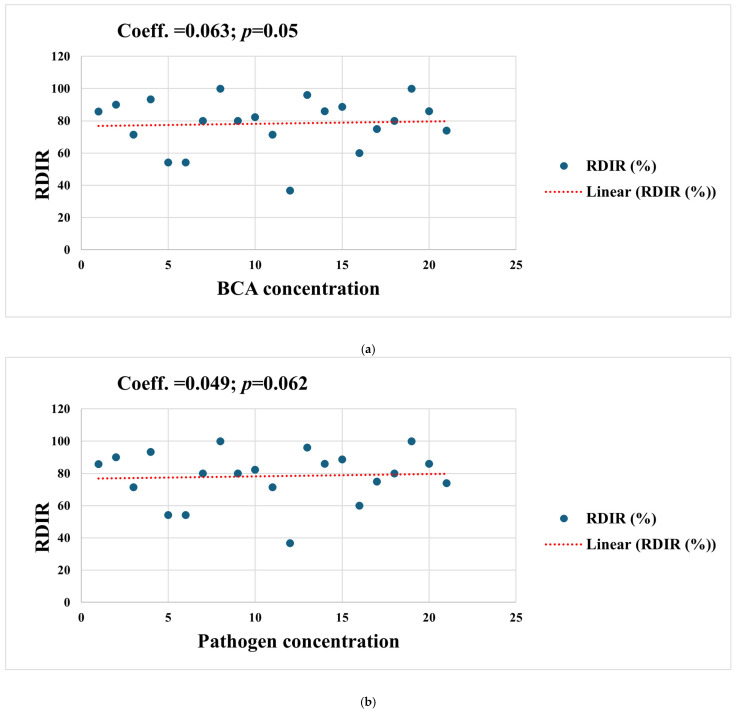

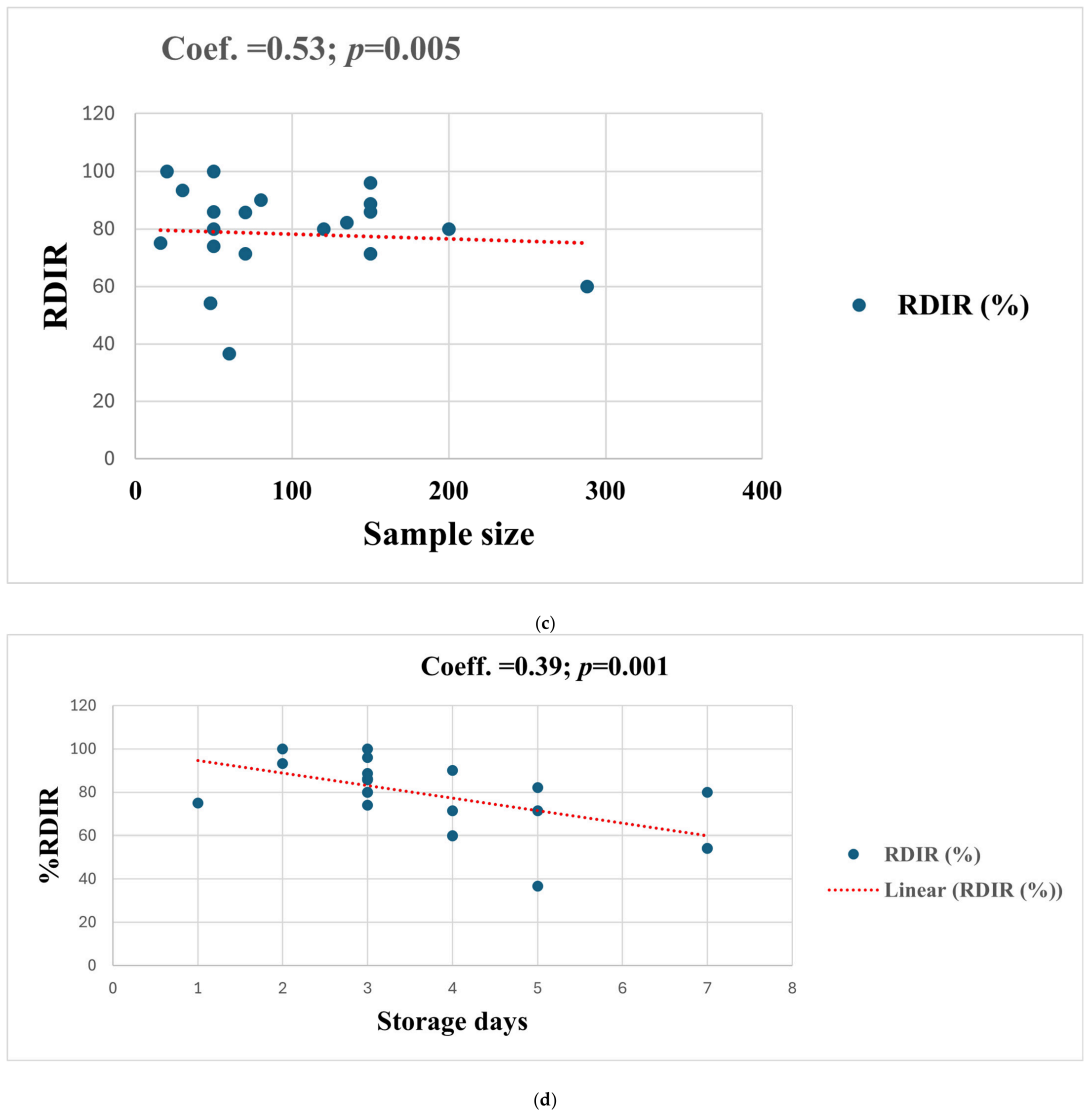

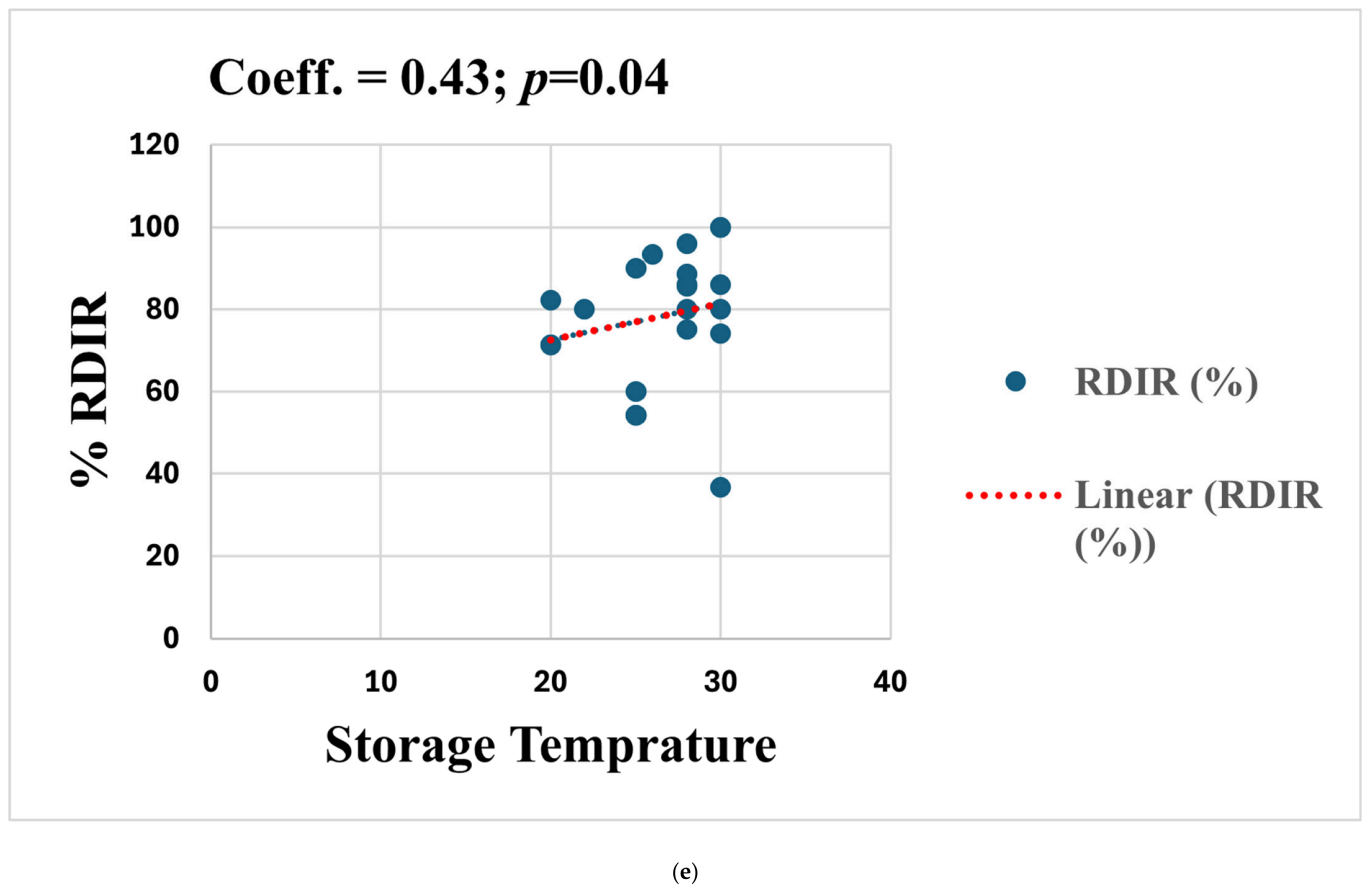
Meta-regression between the means of relative disease incidence reduction (RDIR%) and (**a**) BCA concentration. (**b**) Pathogen concentration. (**c**) Sample size. (**d**) Storage days. (**e**) Storage temperature. The red line represents the linear prediction, and the blue circles represent the number of studies included.

Subgroup meta-analysis revealed that different bacterial antagonists were used as potential biocontrol agents to reduce the incidence of soft rot caused by *P. carotovorum* with varying degrees of efficacy (Figure 5). Also, subgroup meta-analysis was conducted based on the type of crops used among the reviewed studies (Figure 6). Subgroup meta-analysis showed that all the reviewed studies were conducted in 3 countries (Figure 7). Subgroup meta-analysis was also stratified based on study design used, storage temperature, and duration of storage (Figure 8, Figure 9 and Figure 10). Meta-regression was conducted to assess the source of high heterogeneity observed from the meta-analysis results (Figure 11).

## 4. Discussion

Postharvest losses in fruits and vegetables continue to pose a critical threat to global food security, especially in regions like East Asia, where agricultural intensification, humid climate, and inadequate cold-chain infrastructure exacerbate disease pressures. Among these losses, soft rot caused by *P. carotovorum* stands out due to its rapid progression, extensive tissue degradation, and significant economic burden on the fresh produce industry. Despite the growing interest in biocontrol as a sustainable alternative to chemical treatments, no prior systematic synthesis has quantified the regional efficacy of microbial antagonists against *P. carotovorum*-induced soft rot. This systematic review and meta-analysis fill this gap by consolidating available data from East Asia and quantifying the overall and subgroup efficacy of biocontrol agents, thus providing robust evidence to inform both scientific inquiry and practical disease management.

While numerous articles were retrieved, only 14 studies met the criteria for inclusion in this review. The limited research on the relative disease incidence reduction in microbial antagonists against *P. carotovorum* in East Asia, despite its significance and potential risks of causing huge postharvest loss, is concerning. Previous study have highlighted the effect of *P. carotovorum* in eggplant in Xinjiang [3]. All fourteen studies were conducted in three countries: mainly China (studies; 11), South Korea (studies; 2), and Taiwan (study; 1). The uneven geographical distribution of studies with disproportionate representation of other countries from East Asia likely reflects differences in research focus, presence of food science or related institution and laboratory capacity. This findings further demonstrated that China had a greater interest in using different microbial antagonists to control the postharvest decay of fruits and vegetables caused by *P. carotovorum*. This can further be explained by the higher number of studies that investigated the efficacy of different microbial antagonists for controlling postharvest losses of fruits and vegetables caused by *P. carotovorum*. A large number of publications were observed between 2018 and 2025 (Table 1), which might indicate the growth of awareness of government, authorities, and researchers about the use of biocontrol agents to control postharvest decay of fruits and vegetables caused by *P. carotovorum* during these years.

The overall pooled estimate of relative disease incidence reduction (RDIR) by microbial antagonists against *P. carotovorum* in East Asia was 82% (95% CI: 74–88%; I^2^ = 88.2%). This finding indicates a high level of efficacy and supports the potential viability of BCAs as an effective intervention under controlled and context-specific conditions. The magnitude of efficacy observed aligns with prior laboratory and greenhouse studies. For instance, refs. [36,43] also reported over 70% disease suppression by *Bacillus* and *L. paracasei* under controlled conditions, but their findings were localized and lacked broader contextual synthesis. Our meta-analysis strengthens these claims through statistical validation across diverse studies, pathogen strains, crops, and environmental settings (storage days and storage temperature).

Evidence of funnel plot asymmetry was observed based on Egger’s regression test, which may indicate the presence of small-study effects. However, given the substantial between-study heterogeneity (τ^2^ = 7.20), the observed asymmetry may not be solely attributable to publication bias but could also reflect genuine differences in study design, sample size, or methodological approaches. Therefore, the findings should be interpreted with caution.

The substantial heterogeneity observed across studies (I^2^ = 88.2%) likely reflects real differences in study design, crop type, and methodological approaches rather than random variation alone. One important contributor is variation in study design, as studies employing combined in vivo and in vitro techniques reported higher RDIR estimates than those studies reporting a combination of in vitro and greenhouse study methods. Also, crop type represents another major source of biological heterogeneity, as different fruits and vegetables vary markedly in tissue architecture, surface pH, nutrient composition, and normal epiphytic microflora, all of which influence BCA colonization and activity [45]. For instance, crops like tomato and cucumber may permit more rapid pathogen proliferation but also facilitate BCA establishment compared to thick-skinned produce such as eggplant or radish [46]. Additionally, the production of crop-specific antimicrobial compounds (e.g., capsaicin) can differentially affect the viability of pathogens and antagonists [47]. This crop-specific trait modulates the competitive dynamics between BCAs and *P. carotovorum*, contributing to the observed variability in RDIR across studies. Consequently, efficacy estimates derived from one crop should not be uncritically extrapolated to others without host-specific validation.

Study design is a significant contributor to the observed heterogeneity, as the included studies reported diverse experimental systems, including in vitro assays, in vivo fruit tests, greenhouse trials, pot experiments, and field evaluations, each with different levels of environmental control and biological complexity. In vitro studies typically overestimate biocontrol efficacy by eliminating competing microflora and optimizing conditions for antagonist-pathogen interactions [48], whereas greenhouse and field studies introduce variables such as fluctuating temperatures, humidity, UV exposure, and normal microbial communities that can suppress or enhance BCA performance [49]. The absence of standardized experimental protocols across studies, including variations in wounding methods, inoculation techniques, and disease assessment scales, further amplifies between-study variance. Therefore, pooling estimates across different study designs, while statistically permissible, inflates heterogeneity and necessitates cautious interpretation of the global pooled effect size.

Interestingly, the subgroup analysis showed that chitosan-enhanced *L. pentosus* and *L. fallax* [40] showed up to 100% disease incidence reduction conducted on radish under controlled in vivo conditions, suggesting comparatively higher efficacy under the conditions evaluated. This exceptional efficacy may be attributed to the synergistic effect of chitosan [40], a well-known elicitor of plant defense and antimicrobial barrier, and the natural antagonism of lactic acid bacteria [40]. These results are consistent with the work of [50,51,52,53], who documented enhanced postharvest disease control when chitosan was combined with microbial antagonists in tomato. While this finding is promising, the small sample size and absence of field validation warrant cautious interpretation.

Similarly, *P. aeruginosa* exhibited 90% efficacy [35], followed closely by *B. velezensis* (85.7%) [35] and *L. paracasei* WX322 (82.2%) [42]. These BCAs are known to produce a wide range of antimicrobial metabolites, such as lipopeptides, siderophores, and enzymes that can inhibit the growth of postharvest pathogens, disrupt quorum sensing, or compete for niche colonization [54,55,56]. The relatively lower efficacy of *Bacillus subtilis* (55%) [38] may be attributed to variability in strain performance, formulation stability, or host–pathogen interactions, as previously highlighted [57,58].

The meta-analysis also revealed that efficacy varied across crop types. While tomato, Chinese cabbage, and green pepper were commonly used, crops such as eggplant and Pinellia ternata were underrepresented, indicating a need for more host-specific studies. Studies on microbial biocontrol efficacy against *P. carotovorum* in different crops are pertinent, considering the devastating effect it causes on various plants. Differences may influence the heterogeneity in efficacy across crops in tissue architecture, surface microflora, and postharvest physiology. For instance, tomato fruits have thin skins and high water content, making them more susceptible to enzymatic degradation, which might allow antagonists to establish more rapidly and competitively exclude pathogens [59,60].

The meta-regression identified several key factors contributing to heterogeneity, including the concentration of BCA inoculum, storage duration, sample size, and storage temperature. The substantial heterogeneity observed (I^2^ = 88.2%) warrants careful interpretation. This level of heterogeneity is not unexpected in agricultural meta-analyses, where studies vary widely in host species, BCA used, storage conditions, and experimental designs. Importantly, high heterogeneity does not invalidate the meta-analysis but rather indicates that the pooled effect size should not be applied uniformly across all contexts. Our subgroup analyses and meta-regression identified key moderators (BCA concentration, storage temperature, storage duration, and sample size) that explain significant portions of this variability. These findings have critical practical implications. Higher BCA cell concentrations generally resulted in better postharvest disease control, affirming the dose-dependent activity of microbial antagonists [61]. Research has shown that it is essential to know the optimal colonization densities for successful postharvest pathogen suppression [62]. Likewise, storage temperature significantly influenced postharvest disease outcomes, underscoring the interaction between abiotic stress and biocontrol efficacy [63]. Notably, storage under lower temperatures may decrease both pathogen proliferation, ripening, and BCA metabolism, thereby slowing the rate of postharvest decay [64].

Interestingly, pathogen inoculum concentration did not significantly affect the efficacy outcomes, suggesting that BCAs remain effective across a range of pathogen pressures. This robustness enhances their utility in real-world applications where contamination levels may vary unpredictably. However, further mechanistic studies are needed to elucidate whether antagonists act primarily through direct inhibition or through host-mediated resistance under such varying inoculum loads.

Evidence of funnel plot asymmetry was observed based on Egger’s regression test, suggesting the presence of small-study effects. This indicates that smaller studies may have reported disproportionately higher efficacy estimates, which could inflate the overall pooled effect size. However, in the context of substantial heterogeneity, funnel plot asymmetry may also arise from true differences in study design, experimental conditions, and biological systems rather than publication bias alone. Consequently, the potential influence of small-study effects should be considered when interpreting the magnitude of the pooled estimate.

Finally, this systematic review and meta-analysis provides evidence that microbial antagonists, particularly chitosan-enhanced *L. pentosus*, *P. aeruginosa*, *B. velezensis*, and *L. paracasei*, have the potential to reduce postharvest soft rot under the conditions studied; however, the magnitude of effect may be influenced by small-study effects and substantial heterogeneity, and should therefore be interpreted with caution. However, knowledge gaps persist in underrepresented countries, less-studied crops, and long-term field validation. Future research should prioritize formulation improvements, the combined use of multiple BCAs, and integration into broader postharvest handling protocols. Scaling up these sustainable alternatives holds promise for improving postharvest quality, reducing economic losses, and minimizing chemical residues in fresh produce.

## 5. Strengths and Limitations

This systematic review and meta-analysis represent the first comprehensive synthesis of the biocontrol efficacy of microbial antagonists against *P. carotovorum*-induced soft rot in fruits and vegetables within East Asia. A major strength of this study lies in its rigorous methodological framework, which adhered to PRISMA guidelines and employed systematic search strategies across multiple reputable databases, including PubMed, Scopus, and Web of Science. The inclusion of studies spanning diverse crops, experimental conditions, and biocontrol agents provides a broad overview of current evidence within the region. Furthermore, the application of subgroup analysis and meta-regression enabled the identification of key factors influencing biocontrol efficacy, including microbial antagonist concentration, storage temperature, and storage duration.

Another strength is the use of a random-effects meta-analytic framework based on Restricted Maximum Likelihood (REML), which accounts for both within- and between-study variability and provides a more robust estimate in the presence of substantial heterogeneity. The incorporation of sensitivity analyses further supports the stability of the findings. Additionally, the assessment of small-study effects using funnel plots and Egger’s regression test enhances the transparency of the analysis.

Despite these strengths, several important limitations should be considered. First, substantial heterogeneity was observed across studies (I^2^ = 88.2%), reflecting considerable variability in experimental designs, host crops, microbial strains, and environmental conditions. Although subgroup and meta-regression analyses identified several contributing factors, a large proportion of heterogeneity remains unexplained, limiting the generalizability of the pooled estimate. Consequently, the overall effect size should be interpreted as a context-dependent summary rather than a universally applicable estimate.

Second, multiple effect sizes were extracted from individual studies where different crops or treatment conditions were evaluated. While this approach increases the amount of usable data, it introduces potential within-study correlation that may influence the precision of the pooled estimates.

Third, evidence of funnel plot asymmetry based on Egger’s regression test (*p* < 0.05) suggests the presence of small-study effects, with smaller studies tending to report higher efficacy estimates. Although this may partly reflect true variability across experimental conditions, the potential for publication bias or selective reporting cannot be excluded.

Fourth, the geographic distribution of included studies was highly uneven, with a predominance of studies conducted in China. This limits the representativeness of the findings across the broader East Asian region and highlights the need for more research in underrepresented countries such as Japan, South Korea, and Taiwan.

Fifth, most studies were conducted under controlled laboratory or greenhouse conditions. While these environments provide valuable mechanistic insights, they may overestimate efficacy compared to real-world postharvest systems, where environmental variability, native microbiota, and logistical constraints play significant roles. The limited number of field and commercial-scale studies restricts the external validity of the findings.

Finally, the relatively small number of eligible studies and effect sizes reduces statistical power, particularly within subgroup analyses where some categories contained fewer than three observations. As such, subgroup-specific findings should be interpreted cautiously. Overall, the pooled estimate is best viewed as a provisional summary of current evidence, and future meta-analyses incorporating a larger and more diverse evidence base will be required to derive more definitive conclusions.

## 6. Conclusions

This systematic review and meta-analysis provide evidence that microbial antagonists have the potential to reduce the incidence of *Pectobacterium carotovorum*-induced soft rot in fruits and vegetables across East Asia, with a pooled relative disease incidence reduction of 82% (95% CI: 74–88%). However, the very high heterogeneity (I^2^ = 88.2%) observed across the included studies indicates that this estimate should be interpreted with considerable caution and viewed as a context-dependent summary rather than a universally generalizable effect size. A total of 14 studies contributing multiple effect sizes were included, reflecting variability in crops, experimental conditions, and treatment applications.

Subgroup analyses suggest that certain antagonists, particularly chitosan-enhanced *Lactobacillus pentosus* (reported as 100% reduction in a single study) and *Bacillus velezensis* (85.7%), may demonstrate comparatively higher efficacy. However, these findings are based on limited data and predominantly controlled-environment experiments and should therefore be considered preliminary pending further validation. Meta-regression identified microbial antagonist concentration, storage temperature, and storage duration as significant moderators, underscoring the importance of application conditions in determining efficacy outcomes.

The findings indicate that microbial antagonists represent a promising component of integrated postharvest disease management strategies and may offer a more environmentally sustainable alternative to synthetic bactericides under certain conditions. However, the presence of substantial heterogeneity and evidence of small-study effects suggests that the magnitude of the observed effect may be overestimated. In addition, the current evidence base is limited by the relatively small number of studies, the geographic concentration of research in China, and the predominance of laboratory and greenhouse experiments over field-based validation.

Consequently, broad-scale recommendations for replacing conventional chemical controls with microbial biocontrol agents would be premature at this stage. Future research should prioritize well-designed field trials under commercial storage conditions, expand geographical representation across East Asia, and establish standardized protocols for evaluating and applying microbial antagonists. Until such evidence becomes available, microbial antagonists are best regarded as complementary tools within integrated pest management frameworks rather than standalone replacements for existing control strategies.

## Figures and Tables

**Table 1:** Study characteristics and relative disease incidence reduction in microbial BCA against *P. carotovorum* in East Asia.

Reference	Events	Sample Size	RDIR (%)	Crop Type	Country	Study Design	Storage Temperature	Storage Days	BCA Types	Pathogen Concentration (cfu/mL)	BCA Concentration (cfu/mL)
[34]	60	70	85.71	Eggplant	China	In vitro and In vivo	28	3	*Bacillus velezensis*	1 × 10^6^	1 × 10^8^
[35]	72	80	90	Pinellia ternate	China	In vitro and In vivo	25	4	*Pseudomonas aeruginosa*	1 × 10^3^	1 × 10^5^
[36]	50	70	71.42	Green pepper	China	In vitro and In vivo	20	4	*Bacillus subtilis*	1 × 10^6^	1 × 10^9^
[37]	28	30	93.33	Kimchi Cabbage	South Korea	In vitro and In vivo	26	2	*Bacteriophage*	1 × 10^8^	1 × 10^8^
[38]	26	48	54.2	Chinese cabbage	South Korea	Green house and in vitro	25	7	*Bacillus subtilis*	1 × 10^6^	1 × 10^8^
[38]	26	48	54.2	Lettuce	South Korea	Green house and in vitro	25	7	*Bacillus subtilis*	1 × 10^6^	1 × 10^8^
[39]	96	120	80	Calla lily	China	Pot experiments and in vitro	22	7	*Myxococcus xanthus*	1 × 10^11^	1 × 10^9^
[40]	20	20	100	Radish	Taiwan	In vitro and In vivo	30	2	*Chitosan enhanced Lactobacillus*	1 × 10^8^	1 × 10^4^
[41]	160	200	80	Chinese cabbage	China	In vitro, Invivo and field	28	3	*Bacillus velezensis*	1 × 10^8^	1 × 10^8^
[42]	111	135	82.22	Green pepper	China	In vitro and In vivo	20	5	*Lactobacillus paracasei*	1 × 10^8^	1 × 10^8^
[43]	107	150	71.33	Green pepper	China	In vitro and In vivo	20	5	*Lactobacillus paracasei*	1 × 10^4^	1 × 10^8^
[1]	22	60	36.67	Green pepper	China	In vitro and In vivo	30	5	*Green pepper fermentation*	1 × 10^8^	1 × 10^6^
[31]	144	150	96	Chinese cabbage	China	In vitro and In vivo	28	3	*Trichoderma asperellum*	1 × 10^7^	1 × 10^7^
[31]	129	150	86	Cherry tomatoes	China	In vitro and In vivo	28	3	*Trichoderma asperellum*	1 × 10^7^	1 × 10^7^
[31]	133	150	88.67	Carrot	China	In vitro and In vivo	28	3	*Trichoderma asperellum*	1 × 10^5^	1 × 10^7^
[31]	173	288	60.1	Tomatoes	China	In vitro and In vivo	25	4	*Bacillus amyloliquefaciens*	1 × 10^5^	1 × 10^7^
[44]	12	16	75	Cucumber	China	Green house and In vitro	28	1	*Paenibacillus peoriae*	1 × 10^8^	1 × 10^6^
[10]	40	50	80	Chinese cabbage	China	In vitro and In vivo	30	3	*Lactobacillus paracasei*	1 × 10^2^	1 × 10^2^
[10]	50	50	100	Cucumber	China	In vitro and In vivo	30	3	*Lactobacillus paracasei*	1 × 10^2^	1 × 10^2^
[10]	43	50	86	Tomatoes	China	In vitro and In vivo	30	3	*Lactobacillus paracasei*	1 × 10^2^	1 × 10^2^
[10]	37	50	74	Green beans	China	In vitro and In vivo	30	3	*Lactobacillus paracasei*	1 × 10^2^	1 × 10^2^

## Data Availability

No new data were created or analyzed in this study. Data sharing is not applicable to this article.

## Supplementary Materials

The following supporting information can be downloaded at: https://www.mdpi.com/article/10.3390/foods15091580/s1, Supplementary S1: PRISMA Checklist; Supplementary S2. Study characteristics, relative disease incidence reduction of microbial BCA against *P. carotovorum* in East Asia.
