# Supplementary material for: Biocontrol Efficacy of Microbial Antagonists Against Pectobacterium carotovorum-Induced Soft Rot in Fruits and Vegetables in East Asia: A Systematic Review and Meta-Analysis
Source: Foods. 2026 May 4;15(9):1580. doi: 10.3390/foods15091580 (PMC13163911; doi:10.3390/foods15091580)
Supplement: Supplementary file 1 [file foods-15-01580-s001.zip › foods-4242954-supplementary.pdf]

Supplementary S1: PRISMA Checklist

Section and Topic	Item #	Checklist item	Location where item is reported
**TITLE**			
Title	1	Identify the report as a systematic review	Page 1
**ABSTRACT**			
Abstract	2	Abstract	Page 1
**INTRODUCTION**			
Rationale	3	Describe the rationale for the review in the context of existing knowledge.	Page 2
Objectives	4	Provide an explicit statement of the objective(s) or question(s) the review addresses.	Page 3
**METHODS**			
Eligibility criteria	5	Specify the inclusion and exclusion criteria for the review and how studies were grouped for the syntheses.	Pages 4
Information sources	6	Specify all databases, registers, websites, organizations, reference lists, and other sources searched or consulted to identify studies. Specify the date when each source was last searched or consulted.	Page 3
Search strategy	7	Present the complete search strategies for all databases, registers, and websites, including any filters and limits used.	Page 3
Selection process	8	Specify the methods used to decide whether a study met the review's inclusion criteria, including how many reviewers screened each record and each report retrieved, whether they worked independently, and, if applicable, details of automation tools used in the process.	Page 4
Data collection process	9	Specify the methods used to collect data from reports, including how many reviewers collected data from each report, whether they worked independently, any processes for obtaining or confirming data from study investigators, and, if applicable, details of automation tools used in the process.	Page 4
Data items	10a	List and define all outcomes for which data were sought. Specify whether all results that were compatible with each outcome domain in each study were sought (e.g., for all measures, time points, analyses), and if not, the methods used to decide which results to collect.	Page 4
	10b	List and define all other variables for which data were sought (e.g., participant and intervention characteristics, funding sources). Describe any assumptions made about any missing or unclear information.	Page 4
Study quality assessment	11	Specify the methods used to assess the quality of the included studies, including details of the tool(s) used, how many reviewers assessed each study and whether they worked independently, and if applicable, details of automation tools used in the process.	Page 5, Suppl. 2
Effect measures	12	Specify for each outcome the effect measure(s) (e.g., risk ratio, mean difference) used in the synthesis or presentation of results.	n/a as narrative synthesis
Synthesis methods	13a	Describe the processes used to decide which studies were eligible for each synthesis (e.g., tabulating the study intervention characteristics and comparing against the planned groups for each synthesis (item #5)).	Page 4

Section and Topic	Item #	Checklist item	Location where item is reported
	13b	Describe any methods required to prepare the data for presentation or synthesis, such as handling missing summary statistics or data conversions.	Page 5
	13c	Describe any methods used to tabulate or visually display the results of individual studies and syntheses.	Page 5
	13d	Describe any methods used to synthesize results and provide a rationale for the choice(s). If meta-analysis was performed, describe the model(s), method(s) to identify the presence and extent of statistical heterogeneity, and software package(s) used.	Page 5
	13e	Describe any methods used to explore possible causes of heterogeneity among study results (e.g., subgroup analysis, meta-regression).	Page 5
	13f	Describe any sensitivity analyses conducted to assess the robustness of the synthesized results.	Page 5
Reporting bias assessment	14	Describe any methods used to assess the risk of bias due to missing results in a synthesis (arising from reporting biases).	Page 6
Certainty assessment	15	Describe any methods used to assess certainty (or confidence) in the body of evidence for an outcome.	Page 5
**RESULTS**			
Study selection	16a	Describe the results of the search and selection process, from the number of records identified in the search to the number of studies included in the review, ideally using a flow diagram.	Page 6
Study characteristics	17	Cite each included study and present its characteristics.	Page 9, Table 1
Quality assessment of studies	18	Present assessments of quality for each included study.	Suppl. 2
Results of individual studies	19	For all outcomes, present, for each study: (a) summary statistics for each group (where appropriate) and (b) an effect estimate and its precision (e.g., confidence/credible interval), ideally using structured tables or plots.	Pages 9-13
Results of syntheses	20a	For each synthesis, briefly summarise the characteristics and risk of bias among contributing studies.	Suppl. 2
	20b	Present results of all statistical syntheses conducted. If meta-analysis was done, present for each the summary estimate and its precision (e.g., confidence/credible interval) and measures of statistical heterogeneity. If comparing groups, describe the direction of the effect.	Pages 8-15
	20c	Present results of all investigations of possible causes of heterogeneity among study results.	Pages 13-15
	20d	Present results of all sensitivity analyses conducted to assess the robustness of the synthesized results.	Pages 6-15
Reporting biases	21	Present assessments of risk of bias due to missing results (arising from reporting biases) for each synthesis assessed.	Page 15, Fig 8

Section and Topic	Item #	Checklist item	Location where item is reported
Certainty of evidence	22	Present assessments of certainty (or confidence) in the body of evidence for each outcome assessed.	Page 8-13
**DISCUSSION**			
Discussion	23a	Provide a general interpretation of the results in the context of other evidence.	Pages 16-17
	23b	Discuss any limitations of the evidence included in the review.	Page 17
	23c	Discuss any limitations of the review processes used.	n/a
	23d	Discuss implications of the results for practice, policy, and future research.	Pages 17
**OTHER INFORMATION**			
Registration and protocol	24a	Provide registration information for the review, including register name and registration number, or state that the review was not registered.	n/a
	24b	Indicate where the review protocol can be accessed or state that a protocol was not prepared.	n/a
	24c	Describe and explain any amendments to the information provided at registration or in the protocol.	n/a
Support	25	Describe sources of financial or non-financial support for the review and the role of the funders or sponsors in the review.	Page 18
Competing interests	26	Declare any competing interests of review authors.	Page 19
Availability of data, code, and other materials	27	Report which of the following are publicly available and where they can be found: template data collection forms; data extracted from included studies; data used for all analyses; analytic code; any other materials used in the review.	All relevant data are within the manuscript and supplementary file

From: Page MJ, McKenzie JE, Bossuyt PM, Boutron I, Hoffmann TC, Mulrow CD, et al. The PRISMA 2020 statement: an updated guideline for reporting systematic reviews. BMJ 2021;372:n71. doi: 10.1136/bmj.n71

For more information, visit: http://www.prisma-statement.org/

Supplementary S2. Study characteristics, relative disease incidence reduction of microbial BCA against P. *carotovorum* in East Asia

Authors, Year	Study design	Country	BCA types	JBI 1	JBI 2	JBI 3	JBI 4	JBI 5	JBI 6	JBI 7	JBI 8	JBI 9	Total	Quality
Zhang et al. 2024a	In vitro and In vivo	China	*Bacillus velezensis*	1	1	0	1	1	1	1	1	0	7	High
Dong et al. 2010	In vitro and In vivo	China	*Pseudomonas aeruginosa*	1	1	1	1	1	1	1	1	1	9	High
Feng et al. 2024	In vitro and In vivo	China	*Bacillus subtilis*	1	1	0	1	1	1	1	0	0	6	Moderate
Lee , S,.et al. 2021	In vitro and In vivo	South Korea	*Bacteriophage*	1	1	1	1	1	1	1	1	1	9	High
Lee,S. W.et al. 2014a	Green house and in vitro	South Korea	*Bacillus subtilis*	1	0	1	0	1	1	1	1	0	6	Moderate
Lee,S. W.et al. 2014b	Green house and in vitro	South Korea	*Bacillus subtilis*	1	1	0	1	1	1	0	1	0	6	Moderate
Li et al. 2018	Pot experiments and in vitro	China	*Myxococcus xanthus*	1	1	0	1	1	1	1	1	0	7	High

Lin et al. 2020	In vitro and In vivo	Taiwan	*Chitosan enhanced Lactobacillus*	0	0	0	1	1	1	1	0	0	4	Moderate
Ma et al. 2023	In vitro, Invivo and field	China	*Bacillus velezensis*	1	1	0	1	1	1	0	0	0	5	Moderate
Qi et al. 2021	In vitro and In vivo	China	*Lactobacillus paracasei*	1	0	0	1	1	1	1	1	0	6	Moderate
Tang et al. 2025	In vitro and In vivo	China	*Lactobacillus paracasei*	1	1	0	1	1	0	1	1	0	6	Moderate
Yi et al. 2021	In vitro and In vivo	China	*Green pepper fermentation*	1	1	0	1	1	1	1	1	0	7	High
Zhan et al. 2024a	In vitro and In vivo	China	*Trichoderma asperellum*	1	1	0	1	1	1	1	1	0	7	High
Zhan et al. 2024b	In vitro and In vivo	China	*Trichoderma asperellum*	1	1	1	1	1	1	1	1	1	9	High
Zhan et al. 2024c	In vitro and In vivo	China	*Trichoderma asperellum*	1	1	1	1	1	1	1	1	1	9	High
Zhang et al. 2024e	In vitro and In vivo	China	*Bacillus amyloliquefaciens*	1	0	0	1	0	1	0	0	1	4	Moderate

Zhao et al. 2022	Green house and In vitro	China	*Paenibacillus peoriae*	1	1	1	1	1	1	1	1	1	9	High
Zhu et al. 2021a	In vitro and In vivo	China	*Lactobacillus paracasei*	1	1	0	1	1	0	1	1	0	6	Moderate
Zhu et al. 2021b	In vitro and In vivo	China	*Lactobacillus paracasei*	1	1	1	1	1	1	1	1	1	9	High
Zhu et al. 2021c	In vitro and In vivo	China	*Lactobacillus paracasei*	0	1	1	1	0	1	0	1	1	6	Moderate
Zhu et al. 2021d	In vitro and In vivo	China	*Lactobacillus paracasei*	1	1	0	1	0	1	1	1	0	6	Moderate

not available, Scoring: Yes = 1, No/Unclear = 0. Total score 0–9. Quality tiers: High (7–9), Moderate (4–6), Low (0–3).

The JBI checklist items are:

1. Was the sample frame appropriate to address the target population?
2. Were study participants sampled in an appropriate way?
3. Was the sample size adequate?
4. Were the study subjects and setting described in detail?
5. Was the data analysis conducted with sufficient coverage of the identified sample?
6. Were valid methods used for the identification of the condition?
7. Was the condition measured in a standard, reliable way for all participants?

8. Was there an appropriate statistical analysis?
9. Was the response rate adequate, and if not, was the low response rate managed appropriately?
